# Wheat gluten hydrolysates promotes fermentation performance of brewer’s yeast in very high gravity worts

**DOI:** 10.1186/s40643-020-00355-1

**Published:** 2021-01-07

**Authors:** Huirong Yang, Teodora Emilia Coldea, Yingjie Zeng, Haifeng Zhao

**Affiliations:** 1 College of Food Science and Technology, Southwest Minzu University, Chengdu, 610041 China; 2 Faculty of Food Science and Technology, University of Agricultural Sciences and Veterinary Medicine of Cluj-Napoca, 400372 Cluj-Napoca, Romania; 3 School of Food Science and Engineering, South China University of Technology, Guangzhou, 510640 China; 4 Research Institute for Food Nutrition and Human Health, Guangzhou, 510640 China

**Keywords:** Wheat gluten hydrolysates, Brewer’s yeast, Very high-gravity worts fermentation, Fermentation performance, Physiological parameter

## Abstract

The effects of wheat gluten hydrolysates (WGH) and their ethanol elution fractions obtained on XAD-16 resin on physiological activity and fermentation performance of brewer’s yeast during very-high-gravity (VHG) worts fermentation were investigated. The results showed that the addition of WGH and their elution fractions in VHG worts significantly enhanced yeast biomass and viability, and further increased the fermentability, ethanol yield and productivity of yeast. Supplementation with 40% ethanol fraction exhibited the highest biomass (6.9 g/L dry cell), cell viability, fermentability (82.05%), ethanol titer (12.19%, v/v) and ethanol productivity during VHG worts fermentation. In addition, 40% ethanol fraction supplementation also caused the most consumption of amino acid and the highest accumulation of intracellular glycerol and trehalose, 15.39% of increase in cell-membrane integrity, 39.61% of enhancement in mitochondrial membrane potential (MMP), and 18.94% of reduction in intracellular reactive oxygen species (ROS) level in yeast under VHG conditions. Therefore, WGH supplementation was an efficient method to improve fermentation performance of brewer’s yeast during VHG worts. 
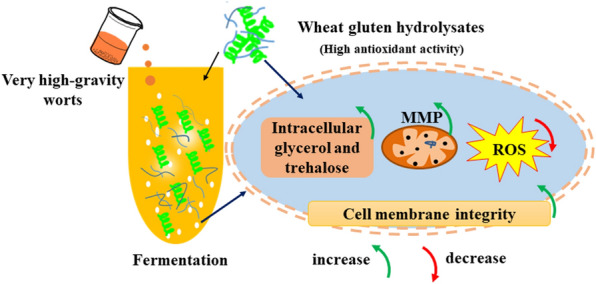

## Introduction

With the development of beer brewing industry, cost and energy savings become especially important to modern brewery. Very-high-gravity (VHG) worts fermentation becomes more and more popular due to its high yield and economy (Lei et al. 2016). However, pitching yeast cells would encounter hyperosmolarity and high ethanol concentration during VHG worts fermentation (Burphan et al. 2018; Yang et al. 2019a). All of these stresses affect not only physiological activity of yeast, but also ethanol production, which would result to a slow or stuck fermentation (Auesukaree 2017; Zhang et al. 2017; Burphan et al. 2018). During VHG worts fermentation, a number of attempts for increasing multiple-stress tolerance in yeast have been carried out to enhance production efficiency (Lei et al. 2016; Zhou et al. 2018). Of these attempts, the addition of nutrients in the medium was shown to be very effective (Auesukaree 2017; Zhao et al. 2017). It has been reported that the addition of amino acids (Lys, Leu and His) and peptides (Lys-Leu) to VHG worts could enhance the biomass, fermentability and ethanol production of yeast cells (Lei et al. 2013b; Yang et al. 2018b). Moreover, previous studies indicated that amino acids (such as Arg, Cys, Met, Pro, Trp and Tyr) and peptides (glutathione) addition could enhance the physiological activity of yeast by improving mitochondria function, reducing intracellular ROS accumulation, and increasing the cell wall and membrane integrity (Shug and Madsen 1994; Albrecht et al. 2000; Burphan et al. 2018; Lim et al. 2018; Zhang et al. 2018).

Wheat gluten as a by-product of wheat starch attracts more and more attention due to its abundant and inexpensive advantages (Agb et al. 2018; Yang et al. 2018a). However, the use of wheat gluten was limited due to lack of some desirable functional properties. Extensive researches have been done to improve the functional properties of wheat gluten by various modification methods (Koo et al. 2014; Liu et al. 2017; Agb et al. 2018; Pietsch et al. 2018). Among these modification methods, enzymatic modification of proteins is gaining much interest due to the various functional properties of hydrolysates (Liu et al. 2017; Agb et al. 2018; Pietsch et al. 2018; Zhou et al. 2018). Wheat gluten hydrolysates (WGH) have been proved to have various bioactivity, such as antioxidant activity, opioid-like activity, immunological activity, and enhanced stress tolerance in yeast (Huebner et al. 1984; Horiguchi et al. 2005; Koo et al. 2014; Yang et al. 2019a). However, the relationships between molecular structure and bioactivity of WGH were totally unclear. Our previous studies also found that the growth, physiological activity and morphology of yeast cells could be improved in the presence of high osmotic pressure and high ethanol concentration by adding WGH (Yang et al. 2018c). However, the effects of WGH supplementations on the physiological characteristics and fermentation performances in brewer's yeast during the VHG worts fermentation were not elucidated thoroughly.

The present study aimed to assess the effects of WGH and their elution fractions obtained on XAD-16 resin additions on physiological activity and fermentation performance of brewer’s yeast during VHG worts fermentation. In addition, the possible mechanisms of improved physiological activity and fermentation performance of yeast cells associated with WGH addition were revealed.

## Materials and methods

### Materials and chemicals

Wheat gluten was purchased from Fengqiu Huafeng Powder Co. Ltd. (Henan, China). Macroporous resins XAD-16 was obtained from H & E Co., Ltd. (Beijing, China). Pancreatin with the activity of 1.2 × 10^5^ U/g was supplied by Novozymes (Novo Nordisk, Bagsvaerd, Denmark). Maltose syrup (60°P) was kindly provided by Shuangqiao Co. Ltd. (Guangzhou, China). All of other chemicals were of the highest commercial grade.

### Yeast strains

The industrial strain used was *Saccharomyces pastorianus* (CGMCC No. 4466) which is deposited in the China General Microbiological Culture Collection Center (CGMCC).

### Enzymatic hydrolysis

A 10% (w/v) aqueous dispersion of wheat gluten was pretreated at 80 °C for 10 min. After cooling, wheat gluten dispersion was adjusted to a pH of 9.0, and incubated in a water bath at 50 °C for 24 h of hydrolysis with 1% Pancreatin addition. The resulting hydrolysates were rapidly cooled to about 25 °C in an ice bath and then centrifuged at 10,000 *g* for 30 min. The supernatant was collected and then lyophilized by an Alpha 1–4 LD plus freeze-dryer (Martin Christ, Germany).

### Preparation of WGH fractions with XAD-16 resin

The lyophilized hydrolysate was dissolved in deionized water to make 40% (w/v) concentration, and then anhydrous ethanol was added into the mixture to reach an ethanol concentration of 80%. Finally, the solution was precipitated at 4 °C, and the supernatant (WGH-A) was obtained by centrifugation at 10,000 *g* for 10 min. The WGH-A was then isolated by elution with 0, 20, and 40% (v/v) ethanol, respectively, on a XAD-16 macroporous resin column (Yang et al. 2018c). The water-washed fraction (WGH-B), 20% ethanol fraction (WGH-C) and 40% ethanol fraction (WGH-D) were collected and lyophilized for further use.

### Wort specifications

All-malt worts (12°P) were made at a pilot-scale brewery. The VHG worts (24°P) were prepared by mixing all-malt worts (12°P) with maltose syrup (60°P). All worts were adjusted to pH of 5.5 by lactic acid and were sterilized in the autoclave at 121 °C for 15 min.

### Fermentation conditions

The seed cells were prepared as described method by Lei et al. (2013b). The seed cells obtained by centrifugation at 4 °C and 8000 *g* for 10 min were inoculated in worts at 1.0 × 10^8^ cells/mL.

Worts added with 3 g/L of WGH-A, WGH-B, WGH-C and WGH-D, respectively, were subjected to ferment in 1 L stoppered measuring cylinders using a fermentation lock and a rubber bung statically at 12 °C (Xu et al. 2019).

### Amino acids composition and free amino nitrogen (FAN) analysis

Amino acid analyzer L-8900 (Hitachi, Tokyo, Japan) was used to analyze amino acids composition. The free amino acid (FAA) and hydrolyzed amino acid (HAA) compositions of four WGH samples were measured as reported by Yang et al. (2018a).

Nihydrin colorimetry method described by Xu et al. (2019) was used to examine the levels of FAN for all samples using a Synergy multimode reader (BioTek, UAS) with 570 nm.

### Biomass and viability determination

The biomass was expressed in dry cells weight by drying at 80 °C in oven to constant weight (Yang et al. 2019b). Cell viability was measured by methylene blue staining method as Lei et al. (2013b) described.

### pH, gravity and ethanol titer

The pH of the fermentation broth was measured with a pH meter (FE28, Mettler-Toledo, Swiss). The gravity and ethanol titer of fermenting samples were examined using an Anton Paar Density Meter DMA 5000 M (Anton Paar GmbH, Austria).

The percentage of fermentability is calculated by the following equation:

$$ {\text{Fermentability}}\,{\text{(\% )}} = \frac{{{\text{original}}\,{\text{gravity}}\, - \,{\text{final}}\,{\text{gravity}}}}{{{\text{original}}\,{\text{gravity}}}} \times 100 $$

### Intracellular glycerol and trehalose content

The extraction and analysis of intracellular trehalose and glycerol was performed according to the method of Yang et al. (2018b).

### Measurement of cell membrane integrity, MMP and intracellular ROS

Yeast cells from fermentation liquor were harvested by centrifugation at 4 °C and 8000 *g* for 2 min. The cells were washed three times using PBS and then re-suspended in PBS for further staining. Cell membrane integrity, MMP and intracellular ROS level were examined by propidium iodide (PI), Rh123 (2-[6-amino-3-imino-3Hxanthen-9-yl] benzoic acid methyl ester) and a ROS Assay Kit (Beyotime Biotechnology Co., Ltd, Shanghai, China), respectively. The Rh123 and ROS Assay Kit treated cells were washed and re-suspended with PBS before detection. All the cell samples were performed on a Synergy multimode reader (BioTek, UAS).

### Antioxidant capacity assay

Antioxidant activities of WGH samples were evaluated by ABTS radical cation scavenging activity, oxygen radical absorbance capacity (ORAC) and reducing power according to the methods of Li et al. (2016). The results were expressed as mmol of Trolox equivalents (TE) for 1 mg of WGH samples (mmol TE/g).

### Statistical analysis

All the experiments were performed in triplicates and the results were reported as means ± standard deviation. Statistical calculation of experimental data was performed using SPSS 23 software (SPSS Inc., Chicago, IL, USA) for one-way ANOVA and Duncan’s multiple-range test.

## Results and discussion

### Amino acid compositions of WGH and their ethanol fractions

Physiological and fermentation properties of yeast cells were affected by the composition of nitrogen source during fermentation process (Zhou et al. 2018; Xu et al. 2019). Therefore, the FAA and HAA compositions of WGH-A, WGH-B, WGH-C and WGH-D were measured. As presented in Table 1, WGH-B showed the maximum content of FAA (236.35 mg/g), followed by WGH-A (123.87 mg/g), WGH-C (83.85 mg/g), and WGH-D (8.20 mg/g). However, WGH-B and WGH-D exhibited the lowest and highest HAA contents of 664.33 and 1176.74 mg/g, respectively. Thus, WGH-D contained the highest levels of peptides (approximately 99.03%), followed by WGH-C (91.68%), WGH-A (86.42%) and WGH-B (66.42%). These results further suggested that XAD-16 resin could be used for peptide enrichment from WGH.

**Table 1:** Amino acid compositions of WGH-A and WGH-A elution fractions (mg/g)^a^

Amino acid	WGH-A		WGH-B		WGH-C		WGH-D	
	FAA	HAA	FAA	HAA	FAA	HAA	FAA	HAA
Asp	0.53	21.12	1.05	20.59	0.00	28.80	0.00	14.59
Thr	10.40	19.85	21.07	17.59	2.67	22.85	0.10	21.18
Ser	3.57	34.20	7.17	30.51	0.00	44.80	0.04	31.00
Glu	3.36	404.71	7.35	245.52	0.00	414.80	0.09	615.69
Gly	1.14	23.78	2.50	15.35	0.00	43.60	0.01	24.43
Ala	6.88	21.51	14.08	28.39	0.04	20.25	0.06	10.14
Cys	0.42	4.51	0.65	1.37	0.00	6.09	0.00	6.80
Val	10.08	35.63	20.72	40.57	0.77	38.28	0.29	22.30
Met	4.11	11.14	8.58	14.36	0.00	6.48	0.02	3.59
Ile	9.12	36.17	21.18	37.63	0.00	36.96	0.09	30.97
Leu	22.16	66.71	47.83	79.15	0.00	44.00	0.17	51.58
Tyr	7.88	15.56	20.02	7.29	3.42	14.28	0.56	25.06
Phe	13.65	46.20	10.25	8.14	55.55	91.48	0.22	73.47
Lys	3.65	9.83	8.01	16.29	0.00	4.42	0.03	2.05
NH_4_^+^	5.93	35.95	12.27	30.27	0.26	33.66	0.28	44.35
His	1.73	12.80	3.72	13.68	0.02	12.21	0.01	9.56
Trp	3.64	6.97	0.49	5.92	20.22	24.08	6.14	8.82
Arg	11.11	24.82	23.41	37.60	0.08	12.15	0.09	6.13
Pro	4.53	80.71	5.99	14.14	0.82	108.23	0.00	175.04

^a^WGH-A, supernatant of wheat gluten hydrolysate; WGH-B, WGH-C and WGH-D, 0%, 20% and 40% ethanol elution fractions of WGH-A on XAD-16 resin, respectively

### Antioxidant activity of WGH and their ethanol fractions

As shown in Table 2, four WGH samples exhibited significantly (*p* < 0.05) different ABTS radical cation scavenging activity, ORAC and reducing power. The differences in antioxidant activity of WGH samples might result from different amino acids and peptides in WGH (Li et al. 2016; Zheng et al. 2016). WGH-D with very little FAA content showed the highest ABTS radical cation scavenging activity (0.545 ± 0.019 mmol TE/g), ORAC (1.157 ± 0.036 mmol TE/g) and reducing power (0.262 ± 0.022 mmol TE/g). Therefore, the high antioxidant activity of WGH-D might be attributed to its peptides, but not FAA. Indeed, bioactive peptides extracted from food proteins possessed antioxidant activity (Lorenzo et al. 2018). Meanwhile, previous studies have shown that antioxidant activity of peptides mainly depends on sequence, composition and position of the amino acid as well as spatial conformation and length of the peptide chain (Ma et al. 2018). Thus, the high levels of antioxidant amino acids (Ala, Cys, Met, Tyr, and Trp, 47.63 mg/g) in WGH-D might be responsible for its high antioxidant activity.

**Table 2:** Antioxidant activities of WGH-A, WGH-B, WGH-C and WGH-D^a^

Samples	ABTS (mmol TE/g)	ORAC (mmol TE/g)	Reducing power (mmol TE/g)
WGH-A	0.393 ± 0.012^b^	0.584 ± 0.043^b^	0.198 ± 0.028^a^
WGH-B	0.310 ± 0.014^a^	0.240 ± 0.032^a^	0.161 ± 0.018^a^
WGH-C	0.484 ± 0.025^c^	0.739 ± 0.028^c^	0.148 ± 0.019^a^
WGH-D	0.545 ± 0.019^d^	1.157 ± 0.036^d^	0.262 ± 0.022^b^

^a^Each value is the mean ± standard deviation of triplicate determinations. Means with different letters in the column for each antioxidant activity assay are significantly different. WGH-A, supernatant of wheat gluten hydrolysate; WGH-B, WGH-C and WGH-D, 0, 20 and 40% ethanol elution fractions of WGH-A on XAD-16 resin, respectively

### Effects of WGH additions on the growth, vitality and fermentation performance of brewer’s yeast

**Fig. 1:**
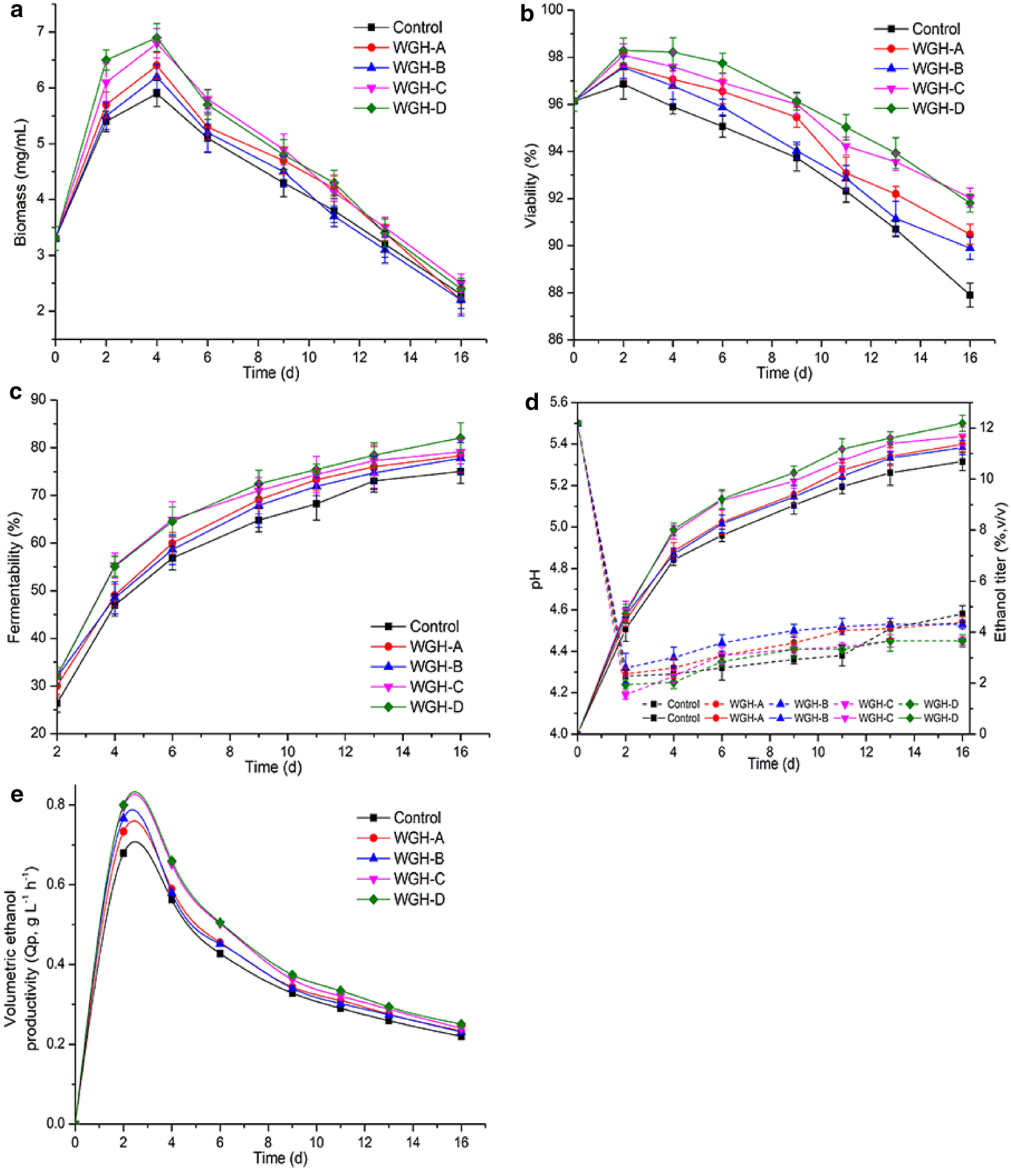
Effects of WGH-A, WGH-B, WGH-C and WGH-D additions on the biomass (**a**), viability (**b**), fermentability (**c**), pH (dashed) and ethanol yield (solid) (**d**) and ethanol productivity (**e**) during VHG fermentation. WGH-A, supernatant of wheat gluten hydrolysate; WGH-B, WGH-C and WGH-D, 0, 20 and 40% ethanol elution fractions of WGH-A on XAD-16 resin, respectively. Error bars indicate the standard deviations from three independent determinations

The effects of four WGH samples on physiological and fermentative property of brewer’s yeast were examined during VHG worts fermentation. As illustrated in Fig. 1a, WGH-A, WGH-B, WGH-C, and WGH-D additions improved yeast cell biomass to various degrees during VHG worts fermentation, which might be due to the different compositions of amino acids and peptides in four WGH samples (Yang et al. 2018c; Zhou et al. 2018). It was noteworthy that WGH-D showed the highest biomass (6.9 g/L dry cell), which might be due to its high content of peptides (99.03%) (Table 1). Previous study found that peptide not only did not compete with the absorption of amino acids, but also could promote the absorption of amino acids (Yang et al. 2018b).

Cell viability is of critical importance to increase ethanol production during VHG worts fermentation (Zhou et al. 2018). As shown in Fig. 1b, supplementations of all four WGH samples could obviously improve cells viability. Similar results had been reported that hydrolysates from soy protein and wheat gluten could improve yeast cell vitality under environmental stresses (Ito et al. 2012; Zhou et al. 2018). Interestingly, WGH-D supplementation exhibited the best cells viability during fermentation (Fig. 1b), which might be due to its active ingredients (amino acids and peptides).

The impact of four WGH samples additions on fermentability of yeast was evaluated, and the results were shown in Fig. 1c. WGH-A, WGH-B, WGH-C, and WGH-D additions showed higher fermentability than the control throughout the fermentation process. By the end of fermentation, worts added with WGH-A, WGH-B, WGH-C, and WGH-D reached 78.31, 77.78, 79.14 and 82.05% of fermentability, respectively, which were higher than the control. These results suggested that supplementing with WGH samples was an effective way to improve fermentability of yeast during VHG worts fermentation. Furthermore, the highest fermentability for the worts added with WGH-D might be attributed to the higher biomass (Fig. 1a) and better cell vitality (Fig. 1b).

pH is a key parameter in fermentation, because it affects the property of cell physiology, and the change of pH could reduce ethanol production under certain circumstances (Peralta-Contreras et al. 2014). The initial pH of the medium was 5.5, and the general trend could be observed in Fig. 1d. The pH values decreased rapidly to a minimum (around 4.2–4.3) during the initial 2 d of VHG worts fermentation, and then increased slowly with the fermentation process. In addition, WGH-A, WGH-B, WGH-C and WGH-D supplements changed the pH of fermentation broth in varying degrees, which might be caused by the changes in nitrogen metabolism and the formation of organic acids (Peralta-Contreras et al. 2014).

As shown in Fig. 1d, ethanol titers in worts supplemented with WGH-A, WGH-B, WGH-C, and WGH-D were 10.69, 11.37, 11.24, 11.68 and 12.19% (v/v), respectively, at the end of fermentation. Worts supplemented with WGH samples showed higher ethanol titer compared to the control, which might be due to the improvement on nitrogen source in worts by WGH samples addition (Zhou et al. 2018). Meanwhile, high ethanol titers were often accompanied by high-fermentation productivity (Deesuth et al. 2016). The addition of WGH-D exhibited the highest ethanol titer (Fig. 1d) and high ethanol productivity (Fig. 1e) during the fermentation process, which might be affected by multiple factors like higher biomass, better cells viability and higher fermentability.

### Effects of WGH additions on nitrogen metabolism of brewer’s yeast

The effects of WGH-A, WGH-B, WGH-C, and WGH-D additions on FAN and amino acid absorption of yeast were evaluated during VHG worts fermentation. The addition of WGH-B in worts exhibited the highest FAN levels (277.75 mg/L), followed by WGH-A (233.67 mg/L), WGH-C (204.62 mg/L) and WGH-D (185.12 mg/L). As Table 3 summarized, the absorption of amino acids in control, WGH-A, WGH-B, WGH-C and WGH-D were 1016.2, 1110.4, 1076.0, 1209.4 and 1212.3 mg/L, respectively. Moreover, higher levels absorption of FAN and amino acids also reflected higher biomass and cell viability (Fig. 1a and b). Meanwhile, the absorption of FAN and amino acids was closely related to fermentability and ethanol titers, and thus could promote fermentation and ethanol production of yeast (Lei et al. 2013a). Although WGH-D contained a very small amount of FAA (8.20 mg/g) (Table 1), it resulted in the maximum absorption of FAN (125.65 mg/L) and amino acids (1212.3 mg/L) (Table 3). These results indicated that bioactive peptides in WGH-D could promote the absorption of amino acids in yeast during the fermentation, which might be due to non-competition uptake between peptide and amino acid (Lei et al. 2013b; Yang et al. 2018b). As outlined in Table 3, addition with WGH-D could observably improve the uptake of Gly, Ile, Leu, Tyr, Phe, NH_4_^+^, His, Trp, Arg and Pro (*p* < 0.05) by yeast cells compared with the control, which further indicated that the addition of peptides could alter the pattern of nitrogen metabolisms in yeast during fermentation (Kitagawa et al. 2008; Yang et al. 2018b).

**Table 3:** Absorption of amino acids in VHG worts fermentation (mg/L)^a^

Amino acid	Control	WGH-A	WGH-B	WGH-C	WGH-D
Asp	72.0 ± 1.8	72.3 ± 2.3	61.9 ± 2.4	72.8 ± 1.6	72.9 ± 1.9
Thr	60.4 ± 2.3	92.0 ± 1.2	123.2 ± 2.6	65.9 ± 1.9	61.8 ± 1.7
Ser	69.9 ± 1.9	80.6 ± 1.4	89.1 ± 1.5	69.4 ± 1.9	71.1 ± 2.2
Glu	61.9 ± 2.6	57.3 ± 1.1	42.9 ± 1.2	65.5 ± 1.3	63.3 ± 1.6
Gly	18.3 ± 0.9	16.0 ± 0.6	16.6 ± 1.1	18.3 ± 0.6	21.4 ± 0.9
Ala	30.9 ± 1.4	47.4 ± 1.9	37.3 ± 1.2	42.7 ± 0.8	57.4 ± 1.0
Cys	1.0 ± 0.2	1.5 ± 0.2	1.70 ± 0.2	0.7 ± 0.1	0.8 ± 0.1
Val	65.0 ± 2.2	46.1 ± 1.4	33.9 ± 1.5	82.4 ± 2.3	100.3 ± 2.6
Met	33.8 ± 1.5	43.8 ± 1.2	45.9 ± 1.3	33.8 ± 1.2	33.9 ± 0.9
Ile	59.7 ± 2.5	69.1 ± 1.5	62.0 ± 1.7	68.9 ± 1.5	71.4 ± 2.1
Leu	148.7 ± 2.4	153.7 ± 2.6	157.0 ± 2.3	155.1 ± 2.7	158.6 ± 2.8
Tyr	51.6 ± 1.0	58.0 ± 1.0	62.3 ± 1.4	65.3 ± 1.9	83.9 ± 2.1
Phe	102.7 ± 2.6	90.1 ± 1.3	79.8 ± 1.9	176.4 ± 2.5	127.9 ± 2.8
Lys	78.6 ± 1.9	89.6 ± 1.9	102.1 ± 2.4	77.9 ± 2.1	79.5 ± 1.9
NH_4_^+^	40.7 ± 1.5	40.3 ± 2.6	34.9 ± 1.7	46.3 ± 1.5	47.2 ± 1.8
His	28.9 ± 1.7	25.1 ± 1.3	20.7 ± 0.6	27.0 ± 0.9	32.7 ± 1.4
Trp	5.0 ± 0.5	12.2 ± 1.4	6.40 ± 0.3	42.6 ± 1.7	23.9 ± 1.2
Arg	82.6 ± 2.6	110.2 ± 1.3	94.1 ± 2.7	95.5 ± 2.3	95.7 ± 1.8
Pro	4.3 ± 0.6	5.1 ± 0.8	4.20 ± 0.5	2.80 ± 0.4	8.60 ± 0.8
Total	1016.2	1110.4	1076.0	1209.4	1212.3

^a^WGH-A, supernatant of wheat gluten hydrolysate; WGH-B, WGH-C and WGH-D, 0, 20 and 40% ethanol elution fractions of WGH-A on XAD-16 resin, respectively

### Effects of WGH additions on the accumulation of trehalose and glycerol in brewer’s yeast

**Fig. 2:**
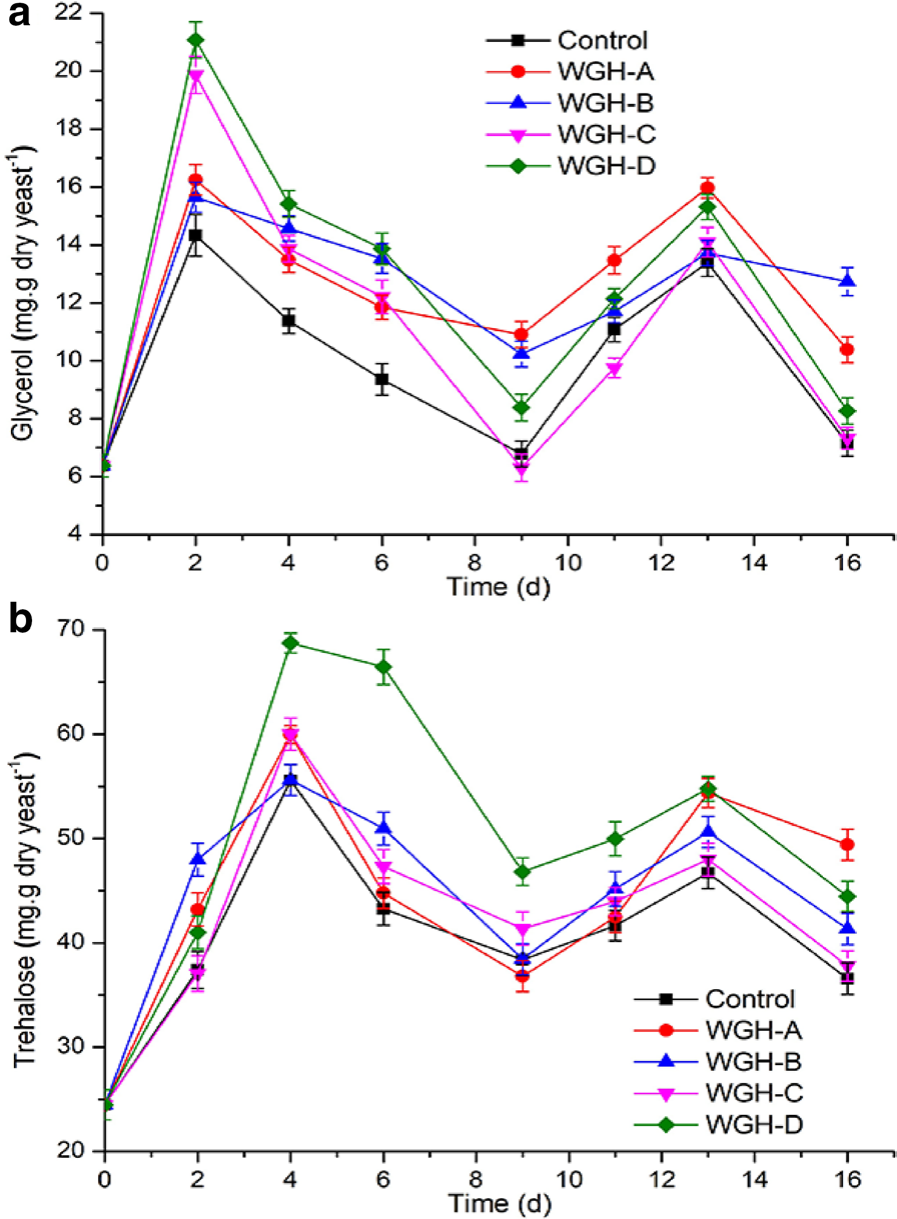
Effects of WGH-A, WGH-B, WGH-C and WGH-D additions on the levels of intracellular glycerol (**a**) and trehalose (**b**) in yeast cells during the VHG fermentation. WGH-A, supernatant of wheat gluten hydrolysate; WGH-B, WGH-C and WGH-D, 0, 20 and 40% ethanol elution fractions of WGH-A on XAD-16 resin, respectively. Error bars indicate the standard deviations from three independent determinations

Glycerol and trehalose, serving as cytoprotective agents, were generated when cells were stimulated by high osmotic pressure, high temperature, and high ethanol concentration (Auesukaree 2017; Luo et al. 2018). Glycerol and trehalose levels in yeast cells during fermentation of worts with WGH samples supplementations were examined, and the results were shown in Fig. 2. The contents of glycerol and trehalose in the cells showed a wave-like change, and peaks appeared in the early and late stages of fermentation, respectively, which might result from high osmotic pressure in the early stage of fermentation and high ethanol concentration in the late stage of fermentation (Yang et al. 2018b). The results suggested that cells could quickly adapt to environmental changes by regulating self-metabolism, such as glycerol and trehalose synthesis (Deesuth et al. 2016; Li et al. 2016; Luo et al. 2018). Compared with the control, WGH samples additions led to markedly higher intracellular glycerol and trehalose levels of yeast cells during VHG worts fermentation. Among them, WGH-D addition led to the highest glycerol (21.06 mg/g dry cell) and trehalose (68.75 mg/g dry cell) levels, with 47.06 and 23.65% of increases compared with the control, respectively. Meanwhile, high trehalose and glycerin levels were closely related to high cell viability (Fig. 1b) and ethanol production (Fig. 1d), and thus the higher contents of glycerol and trehalose were helpful for yeast cells to improve viability and increase ethanol production (Auesukaree 2017; Sethi et al. 2018).

### Effect of WGH additions on the physiological parameters of brewer’s yeast

**Fig. 3:**
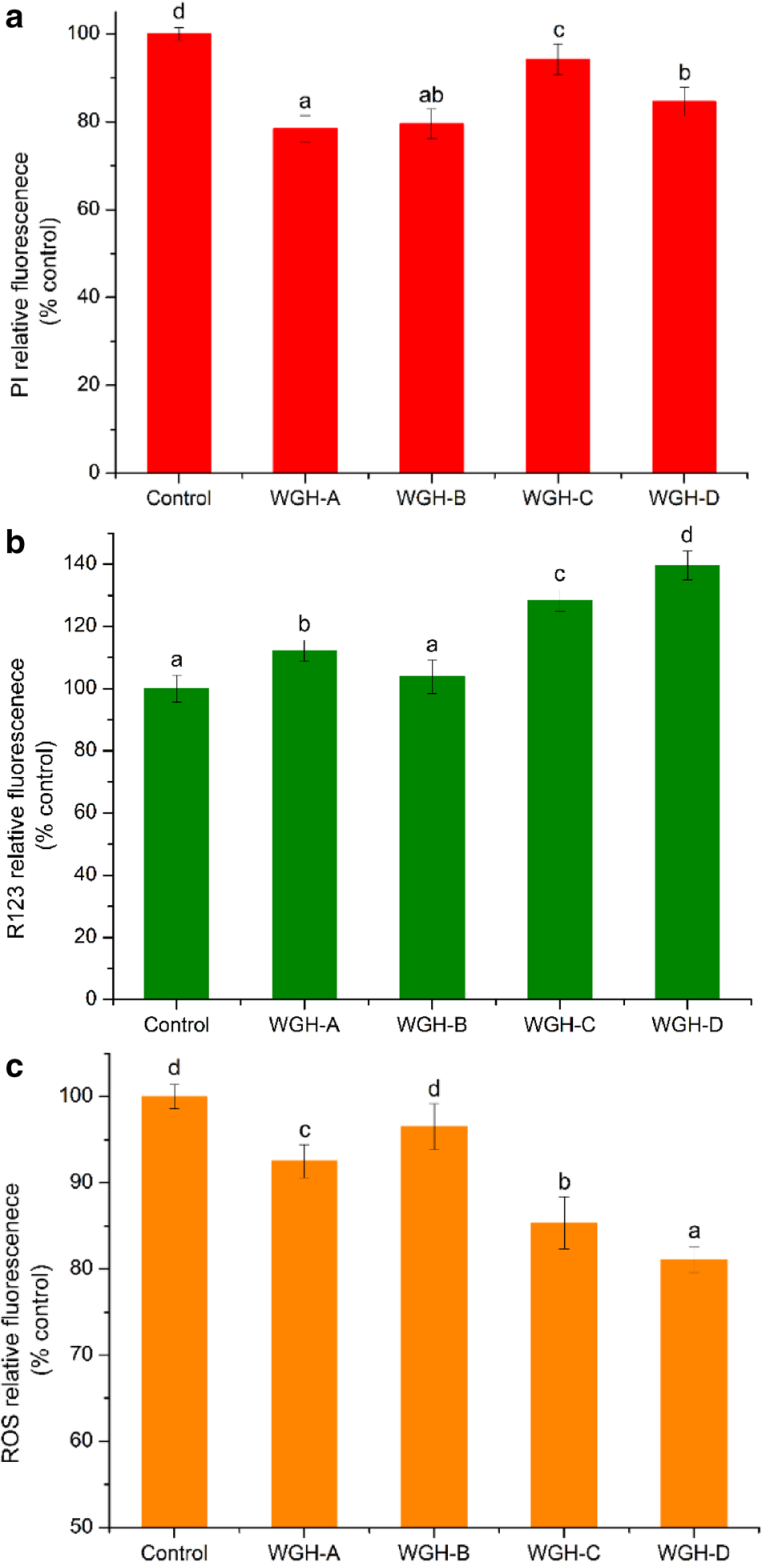
Effects of WGH-A, WGH-B, WGH-C and WGH-D additions on cell membrane integrity (**a**), mitochondrial membrane potential (**b**) and ROS accumulation (**c**) of yeast cells in exponential phase of VHG worts fermentation. WGH-A, supernatant of wheat gluten hydrolysate; WGH-B, WGH-C and WGH-D, 0, 20 and 40% ethanol elution fractions of WGH-A on XAD-16 resin, respectively. a, b, c, d significant differences between control and samples groups at *p* < 0.05

Previous studies have shown that multiple stressors (ethanol, temperature, oxidation and osmotic pressure) led to the reduced cell membrane integrity (Auesukaree 2017; Zhang et al. 2017; Yang et al. 2018a). Therefore, the impacts of WGH-A, WGH-B, WGH-C, and WGH-D on the cell membrane integrity of brewer’s yeast under the VHG worts fermentation were assessed. As shown in Fig. 3a, yeast in worts added with WGH samples displayed lower fluorescence intensity than the control. The reductions of fluorescence intensity in yeast added with WGH-A, WGH-B, WGH-C, and WGH-D reached 21.55, 20.48, 5.78 and 15.37% compared with the control, respectively. These results demonstrated that the WGH supplementations could significantly improve the cell membrane integrity of yeast during the VHG worts fermentation, which might be caused by active substances (amino acids and peptides) in WGH. Furthermore, our previous study showed that active ingredients in WGH could improve yeast cell membrane integrity under high osmotic pressure and high ethanol concentration (Yang et al. 2018a). Thus, the high cell membrane integrity of yeast in the VHG worts added with WGH might be attributed to the absorption of more active substances, such as Gly, Ile, Leu, Met, Arg, Pro and peptides (Forsberg and Ljungdahl 2001; Khalili et al. 2010; Sangeeta et al. 2015; Kalemba and Ratajczak 2018; Pinia et al. 2018; Yang et al. 2018a).

As the power house of cells, mitochondria associated with a variety of cellular activities (Wasiak 2006). Thus, the stability of the MMP played a key role in the physiological property and fermentation performances of yeast (Wasiak 2006; Malina et al. 2018). Figure 3b exhibited the effects of WGH samples additions on the MMP of yeast cells during VHG worts fermentation. Compared with the control, WGH samples additions significantly (*p* < 0.05) increased the fluorescence intensity of yeast cells, which suggested that the WGH samples had the bioactivity of improving MMP for yeast cells under VHG worts fermentation. Similar studies had been reported that essential amino acid (Cys and Gln) and peptides (mitochondria-targeted peptide SS-31 and glutathione) additives had a positive effect on cellular mitochondrial function (Shug and Madsen 1994; Albrecht et al. 2000; Zhao et al. 2005; Gomes et al. 2011; Lim et al. 2018; Zhang et al. 2018). Remarkably, the MMP of yeast cells in WGH-D remained the best, which was 39.61% higher than that in the control. This might be attributable to higher cell membrane integrity and the more absorption of amino acids and bioactive peptides.

Intracellular ROS would generate when the cells were stimulated, such as temperature, high osmotic pressure, strong oxidation, high ethanol concentration (Auesukaree 2017; Zhao et al. 2017, 2019). However, excessive accumulation of ROS led to cell damage. As shown in Fig. 3c, yeast cells in worts added with WGH samples exhibited lower fluorescence intensity than the control, which demonstrated that WGH additions significantly reduced the accumulation of intracellular ROS during VHG worts fermentation (*p* < 0.05). Previous studies had reported that supplements of metal ions, amino acids (Arg, Ala and Pro) or peptide (glutathione) could reduce ROS accumulation and improve yeast tolerance to external pressure (Auesukaree 2017; Yang et al. 2018a). In addition, the reduction of ROS accumulation might also be due to the antioxidant activity of amino acids (Ala, Cys, Met, Trp, and Tyr) and peptides (dipeptide containing Tyr, Trp, Cys and Met, glutathione, proteolytic peptides), which alleviated oxidative damage of yeast cells induced by osmotic stress and high ethanol levels in VHG worts (Sun et al. 2004; Yang et al. 2018a). As shown in Table 1, WGH-D showed the highest antioxidant activity. Meanwhile, WGH-D exhibited the lowest ROS fluorescence intensity (Fig. 3b), with 18.94% of reduction in VHG worts fermentation compared to the control. Therefore, the reduction of ROS accumulation in yeast cells might be partly related to the antioxidant activity of WGH samples. It also should be noted that the low accumulation of intracellular ROS in yeast might due to the joint consequence of higher cell membrane integrity, more stable MMP, and the absorption of more antioxidant amino acids (199.85 mg/L) and bioactive peptides.

## Conclusion

In summary, the additions of WGH and their ethanol elution fractions as nitrogen source to VHG worts significantly enhanced the growth and fermentation performance of brewer’s yeast during fermentation. Improved growth, viability and ethanol fermentation performances of brewer’s yeast in VHG worts with WGH supplementations were related to the increase of intracellular glycerol and trehalose contents, the improvement of cell membrane integrity, the maintenance of MMP, and the decrease of ROS accumulation. These results indicated that WGH as a high-quality nitrogen source could be used to improve beer production efficiency under VHG worts fermentation.

## Data Availability

All data generated or analyzed during this study are included in this article.

## Abbreviations

WGH: Wheat gluten hydrolysates
VHG: Very-high-gravity
MMP: Mitochondrial membrane potential
ROS: Reactive oxygen species
CGMCC: China General Microbiological Culture Collection Center
WGH-A: Wheat gluten hydrolysates supernatant
WGH-B: WGH-A water-washed fraction
WGH-C: WGH-A 20% ethanol fraction
WGH-D: WGH-A 40% ethanol fraction
FAN: Free amino nitrogen
FAA: Free amino acid
HAA: Hydrolyzed amino acid
PI: Propidium iodide
ORAC: Oxygen radical absorbance capacity
TE: Trolox equivalents
